# Improving the hole picture: towards a consensus on the mechanism of nuclear transport

**DOI:** 10.1042/BST20220494

**Published:** 2023-04-26

**Authors:** David Cowburn, Michael Rout

**Affiliations:** 1 Departments of Biochemistry and Systems and Computational Biology, Albert Einstein College of Medicine, Bronx, NY 10461, U.S.A; 2 Laboratory of Cellular and Structural Biology, The Rockefeller University, New York, NY 10065, U.S.A

**Keywords:** cell nucleus, nuclear pores, nuclear protein transportport proteins, nucleic acids

## Abstract

Nuclear pore complexes (NPCs) mediate the exchange of materials between the nucleoplasm and cytoplasm, playing a key role in the separation of nucleic acids and proteins into their required compartments. The static structure of the NPC is relatively well defined by recent cryo-EM and other studies. The functional roles of dynamic components in the pore of the NPC, phenylalanyl-glycyl (FG) repeat rich nucleoporins, is less clear because of our limited understanding of highly dynamic protein systems. These proteins form a ‘restrained concentrate’ which interacts with and concentrates nuclear transport factors (NTRs) to provide facilitated nucleocytoplasmic transport of cargoes. Very rapid on- and off-rates among FG repeats and NTRs supports extremely fast facilitated transport, close to the rate of macromolecular diffusion in cytoplasm, while complexes without specific interactions are entropically excluded, though details on several aspects of the transport mechanism and FG repeat behaviors remain to be resolved. However, as discussed here, new technical approaches combined with more advanced modeling methods will likely provide an improved dynamic description of NPC transport, potentially at the atomic level in the near future. Such advances are likely to be of major benefit in comprehending the roles the malfunctioning NPC plays in cancer, ageing, viral diseases, and neurodegeneration.

## Introduction: structure of a behemoth

**Figure 1.**
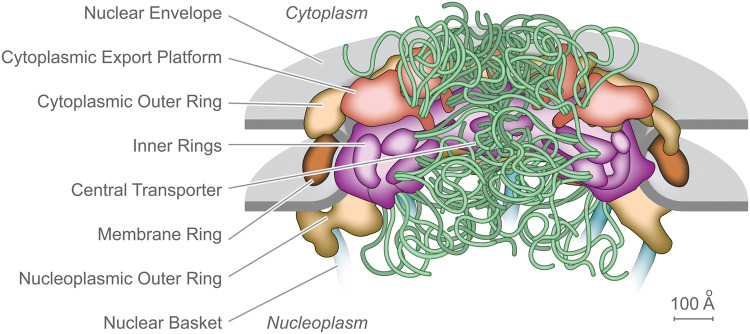
Static representation of an NPC, based on a yeast (*S. cerevisiae*) structure, with the major features labeled [9].

Nuclear pore complexes (NPCs) are among the largest macromolecular assemblies in a eukaryotic cell. NPCs sit in the double-layered nuclear envelope (NE), which provides a barrier separating the nucleoplasm from the cytoplasm. There, NPCs form a platform for the organization of numerous nuclear functions, and critically, act as the sole mediators of macromolecular trafficking into and out of the nucleus. Malfunction of the NPC or its components are linked to many disease states [1–3]. Steady progress has been made in discerning the fine structure of the NPC in yeast and vertebrates, resulting in recent relatively good consensus maps of the NPC's architectural principle features (Figure 1); each NPC is an octagonally near symmetric cylindrical assembly some 100 nm across and 50–100 MDa in mass (depending on species), comprised of ∼500 proteins (termed Nups) representing ∼30 different types that have been fully cataloged for both yeast and vertebrates (reviewed in e.g. [4–6]).

In contrast with this structural understanding, significant areas concerning the dynamic mechanism of the transporting NPC remain undefined. Overall, the dynamic interactions that mediate and regulate transport span about twelve orders of magnitude in time, from nanoseconds to 10 s of seconds. As we need to comprehend this entire scale, no one approach is fully sufficient to give us a complete and unbiased view [7,8]. Here, we discuss why much of the NPC's transport mechanism has proven so refractory to mainstream structural approaches, and how this has led to significant confusion both inside and outside the field, as well as contradictory models representing the full complexity of the structure, dynamics, and biology of the NPC. On the positive side, recent ingenious and orthogonal investigations from multiple groups have begun to overcome prior limitations, and development of exciting new methods is likely to provide major new insights, that already hint that the transport mechanism is perhaps more complex and surprising than previously anticipated.

## The road from static representation to functional understanding

In the nuclear envelope (NE), NPCs are the stationary component of nuclear transport, mediating the mobile phase, comprising the bi-directional traffic of import of proteins to and export of RNAs from the nucleus [9–11]. Much of transport across the NPC is mediated by multiple members of the karyopherin (Kap) family of nuclear transport receptors (NTRs), at rates approaching 1000 molecules/NPC/sec ([12,13] and references therein). Import-Kaps (importins) transport cargos into the nucleus while export-Kaps (exportins) ferry cargos out of the nucleus. Protein cargos are targeted for transport by having a nuclear localization signal (NLS) or export signal (NES). NLSs/NESs bind Kaps, which, in turn, translocate through the NPC, after which the Kap–cargo complex dissociates in its target compartment; their transport directionality is controlled by the nucleotide state of the GTPase Ran, shuttled across the NPC by its dedicated transporter NTF2/p10, a representative of the other major NTR family whose other members, Mex67/NXF1 — Mtr2/NXT1, mediate the export of mRNAs. Other RNAs are exported by cognate Kaps, either directly or via adaptor proteins (and in the case of the 60S pre-ribosomal subunit, also utilizing Mex67/Mtr2) (reviewed in [14]). While small molecules such as metabolites and ions can freely diffuse across the NPC, macromolecules not associated with NTRs cannot pass as efficiently through the NPC, which thus functions as a selective barrier in the CT, leading to the distinction between fast facilitated diffusion (i.e. NTRs/cargoes) and slow or negligible passive diffusion of other macromolecules. While it was previously thought that there was something of a hard upper limit of ∼40 kDa or ∼9 nm radius for this passive diffusion, we now know that there is a power relationship between macromolecular size and the efficiency of its exclusion from the CT [15,16]; which means that few macromolecules access the nucleus through passive diffusion [17]

## The transport mechanism is driven by dynamics and disorder

**Figure 2.**
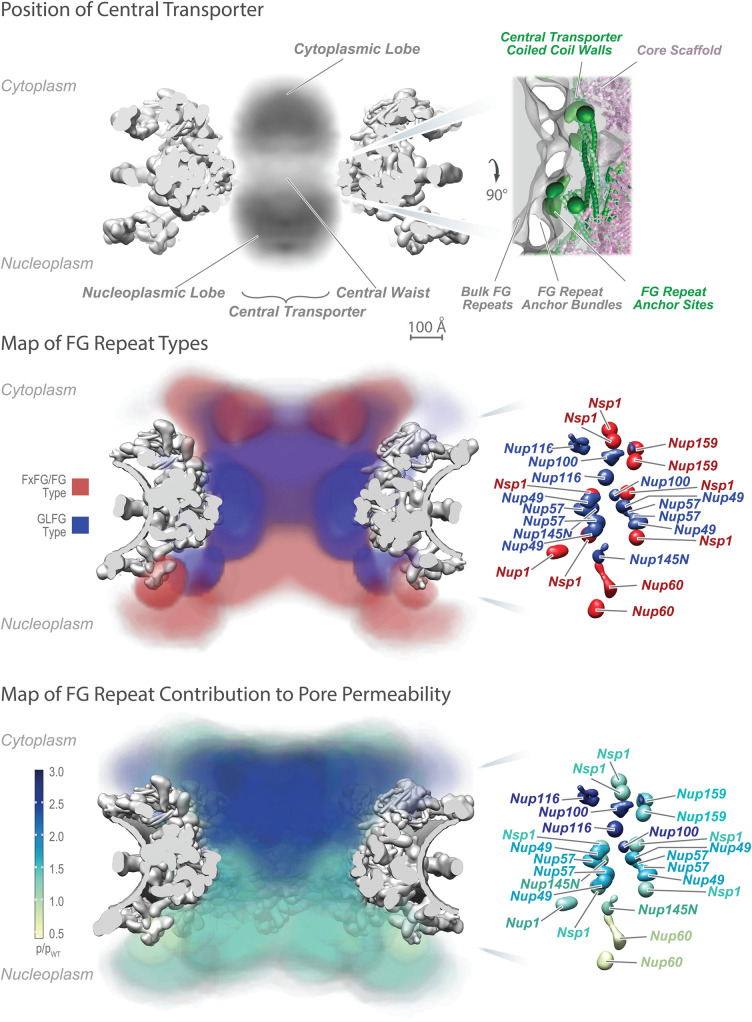
Positions of the FG Nups in the yeast NPC. Upper: Representation of observed electron density of the central transporter within the rigid frame of the NPC. Upper Right: detailed observed density local to the central waist illustrating the FG repeat anchor site. Center: distribution of types of FG repeats within the central transporter based on their mapped anchor sites and inferred by assuming random coil behavior of the FG segments and the absence of NTR/cargoes [15]. Lower: heat map of the contribution of the FG repeat region of each FG Nup to maintaining the passive permeability barrier limiting the passage of nonspecific macromolecules, which appears to be largely maintained by FG Nups enriched in GLFG repeats forming a cap at the cytoplasmic entrance of the NPC [15]. The severity of the permeability defect measured as permeability relative to permeability in wild type, is indicated in increasing shades from minor (light green) to severe (dark blue). Adapted from [18]. The figure is modified form one copyrighted by MPR.

**Figure 3.**
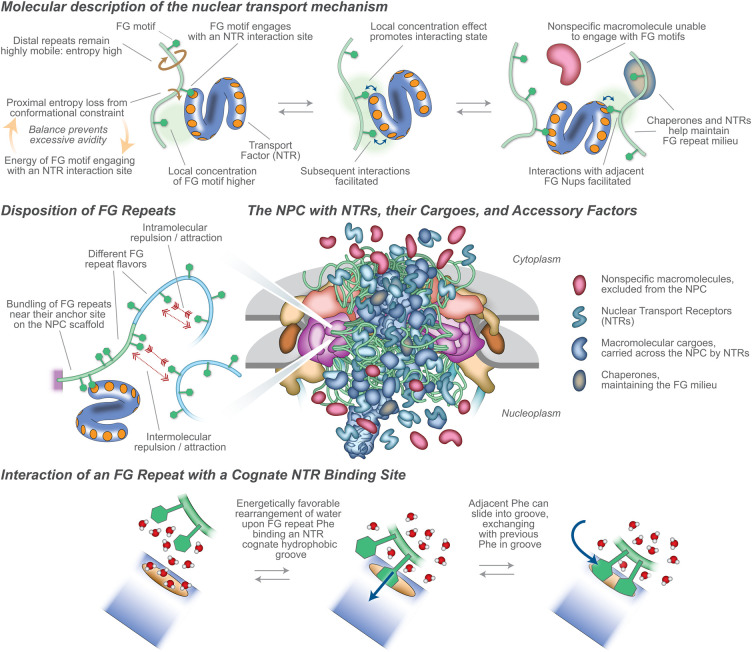
The NPC during nucleocytoplasmic transport. Upper: Molecular representation of the nuclear transport mechanism, at the scale of NTRs and non-specific macromolecules in the proximity of FG repeats. Middle Left: Disposition of FG repeats, showing different potential behaviors. Middle Right: Representation of an NPC with transiting macromolecules, to approximate scale and stoichiometry. Lower: Diagram of two adjacent FG repeats (green) interacting in solvent water (red/white) with a cognate interaction site (orange) on an NTR (blue).

It was originally proposed that the NPC's selective barrier might utilize mechanoenzymes, either by an iris-like gate or motor-driven translocation at the NPC through a permeability barrier of some kind, or that the translocation of NTRs across the NPC was propelled directly by cycles of Ran GTP hydrolysis [19–21]. However, it has been shown that nucleotide hydrolysis is not required for the translocation step across the NPC. Instead, diffusion seemed to be key to trafficking, involving a restriction of passive diffusion and promotion of selective, facilitated diffusion within the CT. An important clue to the transport mechanism came when it was shown that the CT is lined with proteins termed FG Nups [22]. FG Nups are so-called because they contain large intrinsically disordered regions (IDRs) that carry many Phe–Gly (FG) repeats, each separated by ∼20 residues of predominantly hydrophilic linkers (Figures 2 and 3) [11].

Approximately one-third of all Nups contain these regions, which are in the volume of the CT. Changing the dynamic and disordered states of such IDRs is entropically unfavorable, such that any macromolecule attempting to enter their space or push them aside experiences an ‘entropic repulsion’ effect [8,23]. Crucially, it is these FG repeats that were also shown to interact with multiple cognate sites on each NTR and so specifically facilitate its passage across the NPC [24–26]. Based on this information, a ‘virtual gating’ model was proposed in which dynamic multivalent interactions of NTRs with these FG repeats would provide just sufficient avidity to allow their rapid passage across the CT by overcoming entropic repulsion effects of the same IDR regions that otherwise exclude the passage of non-binding, non-specific macromolecules [22,27]. Indeed, it now seems evident that the mechanism for facilitated transport in the CT must include three features: first, the rate of facilitated transport across the NPC is similar to free diffusion within cells [28], so the internal mechanism of facilitated selection must be extraordinarily rapid; second, to maintain facilitated selection, the ratio of concentration of NTRs/cargoes to passive molecules (non-NTRs) within the CT must exceed that external to the CT, i, e, NTRs/cargoes must be relatively concentrated in the CT by interaction with it [9,29–31]; and third, to be consistent with the first and second points, a simple mechanism of inhibition of non-NTR transport is entropic exclusion [10,27,32,33], although exactly how that mechanism plays out in the NPC is still unclear (below). We will now address how these features are produced by the NPC.

## Near to the madding crowd: complexity and crowding within the central transporter generates specificity in transport

Frustratingly, the fact that the CT's component materials are either intrinsically disordered (FG repeats) or extremely heterogeneous (NTRs and their cargoes) has made structure-function studies of it extraordinarily challenging, such that it has been described as ‘structurally elusive and mechanistically controversial’ [7]. Collectively, the anchor sites for the FG repeats in the walls of the CT direct them towards the CT's central axis to generate a highly concentrated and dynamic FG repeat milieu; the anchor sites for most FG repeats are clustered, so that they emanate as bundles near the walls of the CT which can be visualized by electron microscopy, and then merge into a cloud near the CT's axis [9,18] (Figure 2). This generates two organizational features: firstly, the regions of FGs in the bundles near the CT's wall are more diffusionally restricted, as has been indicated *in vivo* [34,35]; and secondly, different kinds of FG repeat (termed ‘flavors’) are at specific positions in the CT's volume (Figure 2). At least in yeast, these flavors can be divided into two broad classes based on an approximate consensus of their Phe-containing repeats and the amino acid composition of the repeat spacers: one of Phe–X–Phe–Gly (FXFG) — like repeats (where X is usually a small hydrophilic amino acid) with hydrophilic spacers often carrying some charged amino acids (Asp, Glu or Lys), and the other of Gly–Leu–Phe–Gly (GLFG) or Phe–Gly (FG) — like consensus repeats spaced by hydrophilic segments of low charge [36]; however, there is considerable variation between FG Nup flavors and even between the same FG Nup homologs of different species [11,37]. One possible reason for such flavor varieties is that they confer different biophysical properties to specific positions in the CT. The role of differences in charged residues in the spacer regions has been suggested to be of significance in the CT by repulsion between like-charged sequences [38] and partitioning of charged and less charged sequences resulting in a permeability barrier [39], but experimental support is lacking. Compelling *in vivo* data also point to the idea that these different flavors and their locations in the CT delineate specific pathways for subsets of NTRs through the NPC [40–48], e.g. it is clear membrane proteins can be actively transported through the NPC in a route distinct from those of soluble cargoes [49,50].

Surprisingly, despite their high concentration the FG repeats do not comprise the majority of the CT — rather, well over three-quarters of the CT at any moment is made of a constant flux of NTRs and their cargoes (see below) [9,18,29,31]. Their sizes vary widely up to many tens of megadaltons, these large cargoes consisting of ribonucleoprotein/mRNA (termed mRNP) cargoes and ribosomal subunit precursors [18,51]. Recently experiments on large cargoes indicated that the increased free energy cost of inserting a large cargo into the dense FG Nup barrier is compensated by the binding to FG Nups via more NTRs per cargo [52], and the very largest cargoes may require expansion of the NPC in some fashion [53,54] consistent with the heterogenous diameters of observed pores [9,55]. This enormous preponderance of NTRs and their cargoes in the CT is the elephant in the room; earlier models have concentrated solely on the roles and states of the FG Nups (below), but in nature, FG repeats in the NPC always exist in the presence of a considerable molar excess of NTRs, meaning that experiments that reconstitute FG repeats in the absence of NTRs could well be examining unnatural states.

We are thus faced with accounting for how these three quarters or more of the CT's mass contributes to its transport behavior, and this realization has led to a recent shift in focus, onto the interplay between FG repeats and NTRs, rather than just the FG repeats alone [56,57]. Crucially, this high concentration of NTRs and cargoes in the NPC, all specifically enriching around the FG repeats, can outcompete and so inhibit nonspecific macromolecular exchange which cannot interact with FG repeats [31,58–60]. Moreover, there is mounting evidence for a slowly exchanging pool of NTRs being maintained at the CT [31,61,62]. Such observations have led to a ‘Kap-Centric’ model, wherein there is a slower exchanging pool of NTRs that are key players in modulating and maintaining the NPC's barrier to non-specific macromolecular exchange [31,63,64]. In summary, ‘the FG Nups are necessary but insufficient for NPC barrier function. NTRs constitute integral constituents of the NPC whose barrier, transport, and cargo release functionalities establish a continuum under a mechanism of Kap-centric control’ [31].

## Fast and furious: active macromolecular transport across the NPC through NTR–FG repeat interactions

A defining characteristic of nuclear transport is the tremendous rates at which each NPC can bidirectionally transport an astonishing variety of cargoes. The family of NTRs is quite large and still may not be fully defined. Remarkably, dozens of different NTRs each mediate separate but often overlapping transport pathways for specific classes of cargoes across the NPC. This multitude of FG interaction sites on each NTR (above; see also Figure 3) again presents the question of how rapid transport avoids slowing by avidity. Generally, the time scales and energies of interactions between FG repeats and NTRs appear very rapid *in vitro* [65,66], and are difficult to measure precisely either *in vivo* or *in vitro* [8]. The specificity of the interactions is clearly linked strongly to the phenylalanyl side chain of FG Nups as revealed by crystallography, NMR, MD simulations, and other methods [67–69]. From solution methods [65,66], and consistent with *in situ* high speed AFM [70] and MD simulation [69], the time scale of each single site NTR–FG interactions is likely of the order of microseconds, so that e.g. ‘weak and ultrafast multivalent Kap–FG interactions allow the Kap–cargo complexes to translocate in a fast and selective manner’ [7]. Atom scale molecular dynamics, supported by NMR data, indicated that the fast exchange between NTRs and individual FG motifs may rely on a sliding-and-exchange mechanism [69], indicating that FG motifs slide on the ample grooves that form NTRs’ binding pockets. This anisotropic sliding may in turn enable fast exchange and rapid facilitated diffusion, such that interacting FG repeats and NTRs exchange particularly rapidly compared with other protein–protein interactions of similar affinity, allowing for the remarkable transport rates observed experimentally (Figure 3).

The emerging picture is that NTRs (with or without cargoes) can transiently skip between FG sites on FG Nups with a low interaction enthalpy dependent on the local concentration of FG Nups and limited in avidity [71] by the entropic motions of the FG Nups’ IDP character [72], while passive diffusion is limited by entropic exclusion, with minimal benefit from interactions with the FG sites. Where the energy for interaction of an F with an NTR pocket comes from is still unclear, but as well as direct amino acid interactions, it seems likely that rearrangement of water molecules from around the hydrophobic F residue and pocket may play a part (Figure 3). A key concept is that the chain mobility of the FG Nups provides entropic exclusion limiting additional enthalpic gains from additional sites. In a detailed specific case, the modest enthalpy of a single interaction of an FG with a given cognate NTR binding pocket (∼−7 kCal/M) is offset by the entropic cost of constraining the FG repeat plus spacer to the NTR's vicinity (∼3 kCal/M). More interactions yield non-linearly more interaction enthalpy, but that is balanced by a similar increase in entropic cost The local concentration effect on enthalpy increasing avidity is limited to 4–6 additional FG sites [72] (Figure 3). One analogy is that the FG Nups form a ‘cloud’ of rapidly diffusing phenylalanine ligands that are constrained to the vicinity of the CT, acting like a ‘solvent’ for the NTR/cargoes with superfast, transient, and weak interactions, although unlike normal solvents the interaction sites are linked (Figure 3). Additional complexity is added by the specificity of interactions between classes of FG Nups and specific NTRs, and by potential interactions (‘cohesion’) among the components.

## FG repeat biophysical behaviors: the quick and the dead?

**Figure 4.**
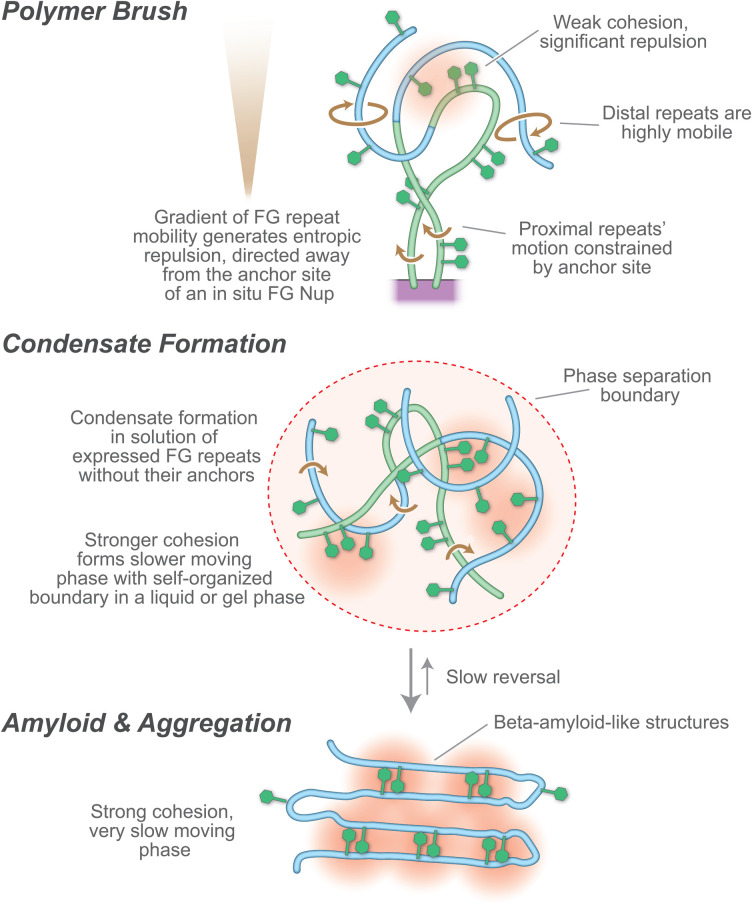
Illustration of the polymorphism associated with FG Nups *in vitro*. Because of their tethering, and lack of tertiary protein structure, the FG Nups are in a polymer brush formation. Controversy centers on whether the brush is entirely intrinsically disordered [65,66], with the degree and speed of motion varying with distance from the tether, and the grafting density (upper) or whether cohesion of the FG repeats results in varying degrees of condensation leading to gels [73,74] (middle) or amyloids [75,76] (lower).

What kind of ‘solvent’ might the FG repeats form in the CT, and in addition to the NTRs’ role, how might its physical states contribute to exclusion of non-specific materials? Biologically, solvents are normally liquids, but the FG repeats cannot form a liquid, because the FG repeats are not freely mobile but rather tethered. As the nature of the barrier to diffusion is hard to discern in NPCs in a cell, various approaches to mimicking the roles of FG Nups have been used. Significantly, selective transport can be mimicked by nanopores of defined composition with FG Nups incorporated using synthetic membranes [58,64,77–80], which demonstrates that the essential phenomenon of selectivity can be reconstructed ex vivo, although providing little information about the state in the CT. However, when isolated as free proteins *in vitro*, FG repeats can assume a range of polymorphisms, from simple intrinsically disordered proteins (IDPs) in solution, to polymer brushes tethered to a surface, to different classes of condensate: liquid–liquid phase separations [73], hydrogels [81], prions [82,83], or rigid amyloid-like gels [75] (Figure 4 and its legend; See Definitions below). These states span a substantial range of viscoelastic properties (see Fig. 4 legend; See Definitions below) [84]. Moreover, all display *in vitro* at least some of the characteristics also seen *in situ*, though none have the performance of native NPCs [80]. This range of FG repeat regions’ states and behaviors is reflected in different proposed models for their function in the CT *in vivo* (reviewed in [39]). But this is putting the cart before the horse — all these states can be manoeuvred to display certain transport characteristics while not addressing what state the FG repeats form in the NPC. So which is the actual state, and speed of FG repeat conformational fluctuations, *in vivo*, remain a subject of investigation and debate.

When IDPs are tethered to a surface, as they are in the CT, they will form a polymer brush (see definitions below), evidence for which is seen for FG repeats *in vivo* [85] and in isolated NPCs [9,18] (Figures 3 and 4). Inclusion of any other macromolecule in the brush is entropically unfavorable as it restricts the entropic thermal motion of the chains in the brush, resulting in an force pushing away from the brush; in the CT, this would result in a kinetic barrier excluding non-specific macromolecules from the pore, these forces scaling with the size of the macromolecule [15,22,27,86]. FG repeats grafted to surfaces (reviewed in [47] [8]) and pores carrying walls grafted with disordered polymers generate such an effective kinetic barrier [87]. While NTRs would also experience (and provide; see above) entropic exclusion, their avidity to the same FG repeats would offset this entropic exclusion and so allow their rapid passage across the NPC [22,27], as quantitatively demonstrated for FG repeats and NTRs *in vitro* [72].

Increasing condensation from freely soluble to rigid gels in these materials is referred to in the field as the result of ‘cohesiveness’ (Figure 4). Note that cohesiveness is used as an omnibus term covering all weak interactions associated with compactness within a single chain, interactions between chains of the same type (homotypic) and interactions with other FG Nups in the vicinity (heterotypic). Based on observations of apparent radius of gyration [36], the GLFG flavor of FG repeats (above) has generally been assigned a higher degree of cohesiveness. In solution this is reflected by GLFG repeats being able to display the complete range of polymorphisms (above) [73,75,76,88]. There is no direct evidence testing which of these states or combination of states is present in the NPC, even though the suggestion that a high degree of condensation (or cohesion) plays a functional role has been widely propagated [74,75,81,88–91]. The nature of the stabilizing cross-links in such gels is also unclear, as is how NTRs reversibly dissolve these links on interactions with F sites. While the suggestion that Phe–Phe interactions may provide such links to form a ‘hydrophobic gel’ [28,74], there is no evidence for significant Phe–Phe interactions in solution or in gel states by NMR [65,73,75,93] or in solution by SANS [94], although such weak interactions are inferred in amyloids of Nup98 by cryo-EM [76].

With the advent of liquid–liquid phase separation (LLPS) in cell biology [95,96], the idea was raised that FG repeats, too, form an LLPS. Indeed, under various conditions, soluble FG repeats can be induced to form LLPSs *in vitro* [90,97–99]. The debate now surrounds: which properties of LLPS the central transporter's FG repeats actually possess? Certainly, LLPSs have two characteristics that FG repeats in the NPC lack, namely, the polymers comprising LLPSs are untethered, and they are defined by their self-organized surface tension [100–102] (See Fig. 4 legend.).

**Definitions of different potential FG repeat states**

**Polymer brush**

‘Polymer brushes are long-chain polymer molecules attached by one end to a surface or interface by some means, with a density of attachment points high enough so that the chains are obliged to stretch away from the interface, sometimes much farther than the typical unstretched size of a chain.’ [103]. Therefore, the CT by definition contains polymer brushes, though additional properties of state may be displayed.

**Condensate**

Dynamic and reversible assemblies of molecules that can dissolve and be reused to perform their function [104]. The usual implication is that the density of a condensate is the result of weak intermolecular forces between the components, and the condensate has significant displacement of solvent from itself. A condensate may be a component of a liquid–liquid separated phase or may be part of a complex structure with components not able to form liquid phases e.g. chromatin [105].

**Gel**

Material with elastic properties, usually with significant permeability to solvent though the gel itself exhibits no steady-state flow and is usually cross-linked. Gels are typically the result of condensates at high concentration [106]. For the NPC, it usually describes *in vitro* formation from GLFG rich FG repeats, and is ascribed without direct evidence to possible function in the CT.

**Liquid–liquid phase separation**

‘Phase separation describes the process by which a well-mixed solution of components de-mixes into two or more coexisting phases with uniform properties. In the simple case of liquid–liquid phase separation, a liquid solution de-mixes into two liquid phases, one dense phase and one dilute phase.’ [104]. In LLPSs, it is solely the surface tension from selective cohesion of its freely mobile components that defines a concentrated compartment [101].

**Viscoelastic complex**

As a result of the formation of some of the above items, the properties of the complexes may be highly variable from essentially fluid, responding to changes of pressure by diffusion, to more gel-like or solid properties, which are elastic, deforming to changes of pressure [107].

## But what is in the central transporter?

Overall, the functional role *in vivo* for changes of state of FG repeats involving LLPS, gels or amyloids is entirely unknown. This issue of structural pleomorphism and its functional implications is a major challenge generally to the structural biology of amyloid and gel-like systems, complicating *in vitro* reconstitution e.g. [108]. Moreover, CT assembly is likely tightly regulated, to balance functional activities against the ageing and aggregation that has been implicated in leading to amyloid like disease states [104]. Indeed, there appear to be active balance and maintenance systems that limit FG repeat condensation *in vivo*. Thus, the observation that removal of highly condensed FG Nups may be facilitated by chaperones [109,110] interacting with FG repeats, suggests that chaperone-like activity may play a role in assembling and maintaining the NPC. Moreover, other work indicates that NTRs contribute actively to preventing aggregation of FG repeats [111], and FG Nup condensate puncta outside the NPC appear to be transient non-essential and even toxic condensates that are absent from healthy cells [112] (also reviewed in [113]), and the more aggregated forms have been associated with disease states e.g. in neurodegeneration [114–116].

Taking all this together, it seems reasonable to suggest that the polymer brush FG repeats of the CT form a ‘restrained concentrate’, where a concentrating effect of FG repeats is achieved by the anchoring of FG Nups in the CT walls, and the density and selective transport behavior of the CT contents results mainly from this constraint plus the recruitment of NTRs/cargoes (above), without necessarily having a *specific* requirement for extensive cohesion to form a gel. This view is most consistent with solution studies of different isolated FG repeat flavors which show a picture of highly mobile, minimally cohesive IDPs [65,66,72,117] with fast, low affinity interactions with NTRs and with very high mobility of tagged FG repeat regions *in vivo* [34,54,85,118]. Notable in this regard, the Lim lab has also contributed key observations that the central portion of the *in situ* CT is dynamic using high speed atomic force microscopy, definitively establishing the movement of the center of the NPC in the 100 ms and faster range and showing that intermingling FG Nups there do not appear to cohere into a highly cross-linked meshwork like an amyloid [35,70]. Some form of weak cohesive forces must exist to some (currently ill-defined) degree; for example, the density and packing of the FG Nups and NTRs/cargoes are subject to the usual dynamic interactions including van der Waals attractive and repulsive forces, complementing specific FG/NTR interactions in which NTRs may bridge between different FG repeats, and other potential specific interactions [39,119,120]. These forces may have some ‘tuning’ role to play in adjusting the permeability and selectivity parameters of the CT.

## Pores in action: a new view *in vivo*

Recent innovative approaches have begun to move the field away from the drawbacks of reliance on only *in vitro* data, to now garnering detailed nanoscale dynamic data on NTRs and FG repeats in the CTs of *in situ* or living NPCs. In particular, the examination of functional FG Nups by measuring fluorescence energy transfer between two neutral fluorophores placed at different positions along the length of FG repeats in the *in vivo* NPC provides a significant advance in our understanding of their dynamic structure [118]. Pioneering advances in design of small amino acid fluorophores, insertion of multiple labeling sites in the appropriate genes, and measurement of the distance distribution of 18 NUP98 segments is consistent with the in-NPC state being close to that of a random polymer in a ‘good solvent’, and is significantly different from the value observed in solution at low concentration that is consistent with a compacted state (as also seen by other methods) [73]. More detailed analysis of the fluorescence lifetime decay using simulations is consistent with extremely rapid polymer motion, and suggestive of some shuffled packing of the FG repeats towards the periphery with a concentration of NTR/cargoes towards the center [118]. Similar new approaches *in situ* and *in vivo* are on the horizon, promising direct observation of transport in action at the molecular level.

Current research reviewed has also concentrated on the function and mechanism of the CT. However, other recent work has pointed to additional factors that may play critical roles in the transport mechanism. These include: a potential role of numerous transport factors in forming the nucleocytoplasmic Ran gradient [57]; accounting for the dilation of the NPC in diffusion control and in response to environmental changes [9,55,56,121,122]; and the existence of multiple and very distinct NPC isoforms, even within the same cell, that may have different transport roles [9,55]. The diversity of NTRs, FG repeats and even NPC isoforms also raises the question as to whether only one general mechanism is employed by the NPC in selective transport and it even seems possible that different NTRs may employ distinct mechanisms, perhaps in concert with specialized NPC isoforms.

## New tools on the block: data integration, modeling and simulation

On their own, these multiple biophysical and cell biological observations are not readily merged into a simple hypothesis of how the CT permits rapid and selective diffusion. Recent modeling papers have focused on the underlying thermodynamic issues of enthalpic/entropic balance in FG Nup/NTR interactions, on the dynamics associated with diffusion, on the roles of cohesion in FG Nup self-interactions, and on the dynamic architecture of the CT and its role in providing specific pathways for diffusion. These both integrate the prior observations and suggest new hypotheses testable by experiment.

While their role *in vivo* is unclear, FG repeat gels are significant as a model for phase separation studies, and an underlying thermodynamic model was recently developed [88] rationalizing the observed increased stability of gels with temperature [73]. The critical limit for gel formation was also calculated for different amino acid compositions using coarse grained modeling at the bead-per-residue level [123]. The effects of cohesiveness on the selective permeability of *in vitro* FG repeat assemblies were simulated over a wide range of cohesiveness, showing that an increase in cohesiveness leads to decreasing permeability but that permeability may be enhanced with weak cohesiveness [124]. Modeling of the FG Nups in the CT in the absence of transport factors suggested a heterogeneous diffusion barrier of several condensates formed by electrostatic pairing rather than FG–FG interactions [39]. The role of the dynamic architecture of the CT was modeled and compared with experimental fluorescence anisotropy data by use of a bead equivalent of 4 nm resolution, and it was proposed that FG repeats are highly mobile and can reptate throughout the CT on timescales similar to experiment [125]. The role of transient formation of voids permitting a size dependent permeation was analyzed [126] using the Onck force field [127]. Regarding modeling passive transport, a Brownian dynamics simulation, with FG repeats represented as spring-like polymer beads and passive diffusing macromolecules as rigid spheres, suggested that the barrier to non-specific diffusion resulted largely from the highly dynamic FG repeats and entropic exclusion [15].

Several recent papers address more directly the modeling of FG repeat's interactions with NTRs. Based on experimental data for FG repeats, NTF2, and non-specific components’ interactions, agent-based modeling [128] discriminated between binding models to discriminate multivalent cases [59]. Crowding by different NTRs affecting interactions with FG Nups were proposed from coarse-grained classical density functional theory application with two residues per bead model for FG Nups, and spheres for NTRs, with the significant conclusion that at high NTR concentrations, there is increased flux [47] consistent with experimental data [129]. Complementing two experimental works on biomimetic nanopores with separate FG Nups [64,77] coarse grained modeling suggested that the NTR Kap95 forms a stable population bound to the CT periphery with fast transport proceeding in the FG-rich central channel.

A fair judgment is that these models are based on a wide range of assumptions/parameters, and that currently their results, in terms of describing details of the transport mechanism, are rather divergent. As the field integrates more data and refines models in the areas of siting of CT components, of parametrization of interactions energies of FG repeats and NTR/cargoes, of increased simulation times comparable to transit times in the NPC, and of comparison to more detailed tracking of individual NTR transits (e.g. [40]), there will hopefully be some convergence towards a realistic representation of the mechanisms of transport. While our knowledge of how FG repeats and NTR/cargoes interact *in vivo* is limited, there is however consensus that ‘stronger interactions and higher concentrations can block the transport. Importantly, accumulation of the transport proteins in the pore can also impede the translocation of inert molecules’ [8]. Similarly, there is agreement that FG repeat/NTR interactions are key to selective diffusion, while there remains controversy in the models about the nature and role of FG repeat cohesive interaction and their role in limiting passive diffusion. A key missing ingredient of current simulations is lack of incorporation of the ‘elephant’ — our updated knowledge of the high density of NTR/cargoes within the CT, and perhaps also limited appreciation of the difficulty of understanding the whole NPC — whose size and detail may require a so-called paradigm shift of approach rather than the cumulative accretion of data [130]

## Perspectives

- Every year, more and more connections are discovered between NPC dysfunction and a range of serious, widespread and challenging human diseases; these include many cancers, neurodegenerative diseases, and a host of viral diseases including most recently SARS-CoV-2 [131,132]. Designing effective therapeutics for such dysregulations depends upon the biomedical community gaining a detailed and comprehensive understanding of all the functionalities associated with NPCs, foremost among these being the mechanisms underlying nucleocytoplasmic transport.
- There is now consensus on many aspects of the mechanisms underlying nucleocytoplasmic transport. FG Nups anchored in the walls of CT generate a brush of intrinsically disordered FG repeat regions that form a high local concentration of FG motifs. NTRs, often carrying cargoes, cross by binding these FG motifs. The Interaction between NTRs and FG repeats leads to a high local concentration of NTRs in the CT, further strongly contributing to competitive exclusion of non-specific macromolecules. Much of the remaining controversy revolves around how non-specific macromolecules are further prevented from crossing the NPC, with possibilities ranging from FG repeats forming slow moving highly cross-linked gels, to weakly (or essentially non-) cohesive FG repeats being highly mobile and entropically excluding only non-interacting macromolecules while facilitating rapid transit of NTRs with cargoes.
- Resolving the remaining controversies, discussed above, is obviously a major priority. We must also come to grips with the astonishing diversity and pliability now being revealed in the NPC's architecture and mechanisms; that different NPCs have different compositions and so may specialize for different transport pathways, that NPCs can change shape in such a way that may modulate transport, and that different cargo types may take different paths across the NPC with different mechanistic details and at different times. Finally, as more mechanistic links between NPCs and diseases are understood, the potential for therapeutic intervention in nucleocytoplasmic transport will likely greatly increase.

## Abbreviations

GLFG: Gly–Leu–Phe–Gly
IDPs: intrinsically disordered proteins
IDRs: intrinsically disordered regions
LLPS: liquid–liquid phase separation
NE: nuclear envelope
NPCs: nuclear pore complexes
NTRs: nuclear transport factors
